# Lateral Extra‐articular Tenodesis With All‐Suture Knotless Anchor

**DOI:** 10.1002/atn2.70345

**Published:** 2026-10-28

**Authors:** Lauren Lucina, Sean Tierney, Ronak M. Patel

**Affiliations:** ^1^ Illinois Center for Orthopaedic Research and Education Westmont Illinois U.S.A.

## Abstract

Although many patients have had satisfactory outcomes from arthroscopic anterior cruciate ligament reconstruction, persistent rotational instability and graft failure still occur. Therefore, the anterolateral ligament complex has been a newer focus of research. The addition of the lateral extra‐articular tenodesis to augment anterior cruciate ligament reconstruction has been found to increase stabilization and reduce rerupture rates. We describe our surgical technique for performing a modified Lemaire lateral extra‐articular tenodesis with an all‐suture knotless anchor.

## Graphical abstract

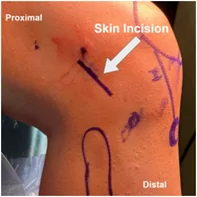

**VIDEO 1:** This video shows the step‐by‐step surgical technique used for lateral extra‐articular tenodesis with Q‐Fix all‐suture knotless anchor in the setting of anterior cruciate ligament repair. An open, lateral view of the right knee in a flexed position is seen throughout the video, and anatomical landmarks are identified. Video content can be viewed at https://doi.org/10.1002/atn2.70345.

The anterolateral ligament complex has been the focus of research in recent years as reconstruction techniques have been devised to augment anterior cruciate ligament (ACL) reconstruction (ACLR). ACL tears are among the most common sports‐related knee injuries, affecting nearly 68.6 per 100,000 individuals annually in the United States.^1^ Although many patients make a full recovery and return to preinjury activity level, postoperative residual rotational instability is persistent in up to 25% to 30% of patients.^1^ A potential contributing factor to the residual rotational laxity is injury to the anterolateral complex, which is predicted by a higher‐grade pivot shift or residual positive pivot shift after ACLR.^1^, ^2^, ^3^

Onggo et al. performed a systematic review and meta‐analysis of 7 randomized controlled trials reporting that the inclusion of lateral extra‐articular tenodesis (LET) to ACLR reduced the rerupture rate by approximately 3‐fold compared with ACLR alone.^4^

Reconstruction of the lateral side provides a secondary restraint stabilization to anterior dislocation and internal rotation of the tibia.^3^ The indications for augmentation have expanded to include revision ACLR, high‐grade pivot shift, ligamentous laxity, and young patients returning to pivoting activities.^5^ Generally, there are 2 reconstruction techniques: (1) anterolateral ligament reconstruction with an autograft or allograft and (2) LET with an iliotibial (IT) band autograft. Multiple biomechanical studies have validated the efficacy of both techniques, allowing surgeons to use their preferred method.^6^, ^7^ We describe our surgical technique through this case‐based approach, performing a modified Lemaire LET with an all‐suture knotless anchor.

## SURGICAL TECHNIQUE

### Preoperative Analgesia and Patient Positioning

The patient is laid supine with the leg of the bed flexed. The patient's ipsilateral knee is placed in a ring knee positioner. The contralateral extremity is placed in the lithotomy position. General anesthesia is given with a local anesthetic. A tourniquet is placed on the ipsilateral upper thigh.

### Surgical Approach and Technique

The LET is performed after the ACLR. The ACL is tensioned and completed, and then, the LET is done.

#### Incision/Exposure

Landmarks are identified and marked along the fibular head, Gerdy's tubercle, and the IT band. With the knee at 90° of flexion, a 3‐ to 4‐cm incision is made over the IT band, centered 3 cm proximal to Gerdy's tubercle (Figure 1). The soft tissue is then traversed with electrocautery. The IT band is exposed circumferentially. Careful dissection is made to avoid disturbing the lateral inferior genicular artery. The underlying exposure is created wide enough to allow for a mobile window 8 to 10 cm proximal to Gerdy's tubercle, providing access to all parts of the IT band (Figures 2 and 3).

**FIGURE 1:**
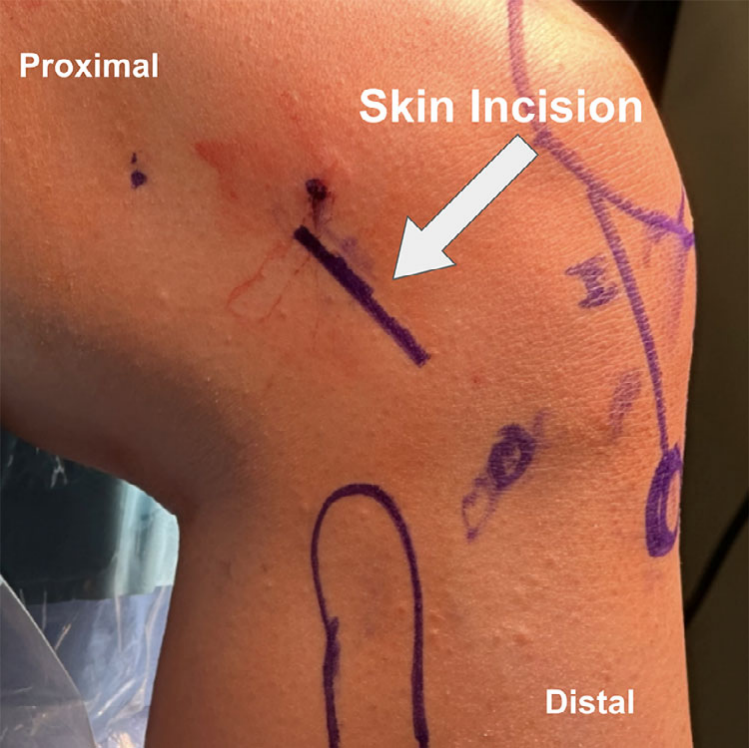
A lateral view of the right knee is shown with the patient in supine position and knee flexed to about 90°. The fibular head, Gerdy's tubercle, and IT band are identified and marked with a marking pen. A 3‐4‐cm skin incision is also identified over the IT band and is centered 3 cm proximal to Gerdy's tubercle. (IT, iliotibial.)

**FIGURE 2:**
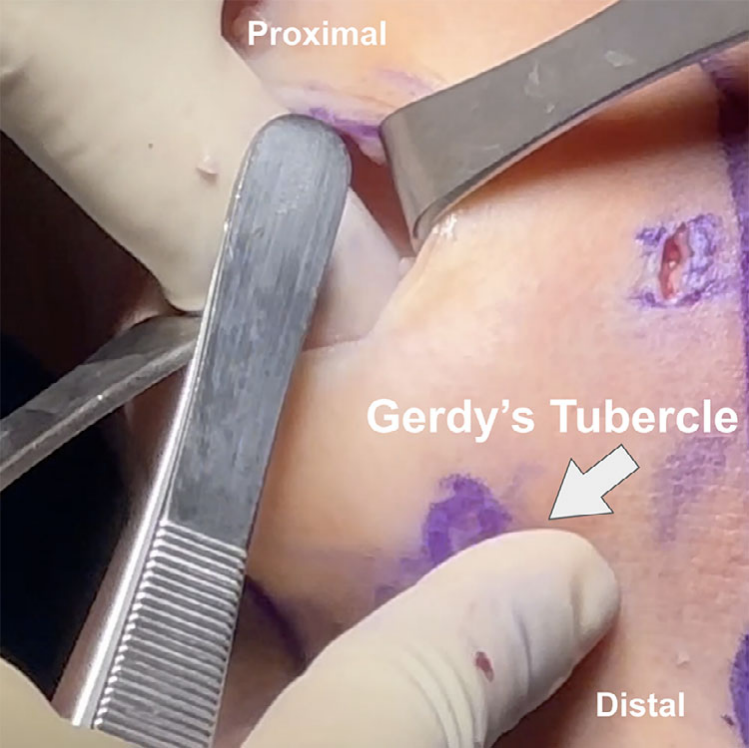
An open, lateral view of the right knee flexed to about 30°. Careful dissection is made to create a mobile window which exposes the IT band distally all the way to Gerdy's tubercle. This is confirmed with palpation before proceeding. (IT, iliotibial.)

**FIGURE 3:**
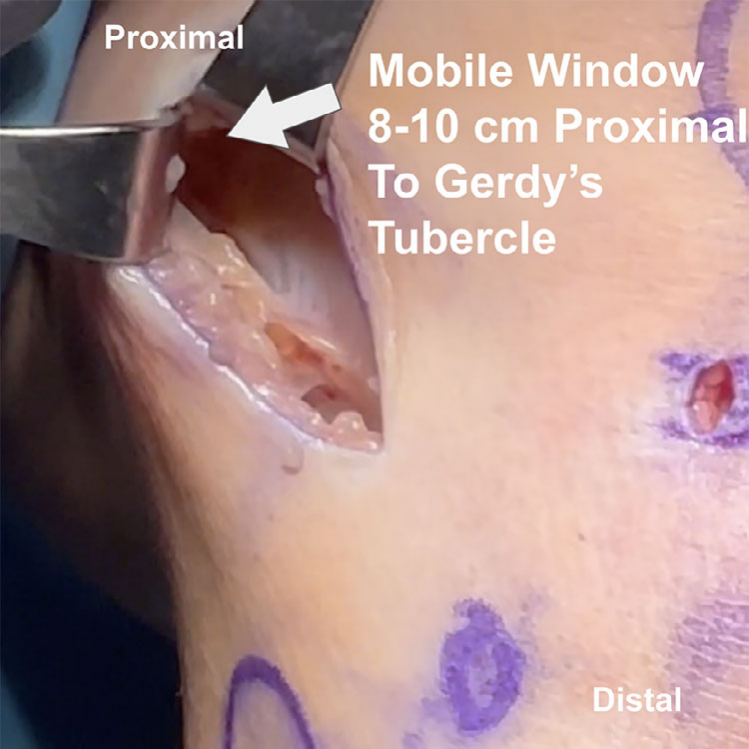
An open, lateral mobile window is created 8‐10 cm proximal to Gerdy's tubercle for direct visualization of the IT band. (IT, iliotibial.)

#### Harvest of IT Band

The anterior and posterior borders of the IT band are identified. Then, measure 1 cm of the central portion of the IT band before harvesting. Use a 15 blade for harvesting to protect the deep structures such as the lateral collateral ligament (LCL), the popliteus tendon, and the underlying capsule (Figure 4). The IT band is then measured with a ruler before being transected at the proximal end. An Allis clamp is placed on the proximal end of the IT band graft, whereas the underlying capsular attachments are cleared with Metzenbaum scissors (Figure 5).

**FIGURE 4:**
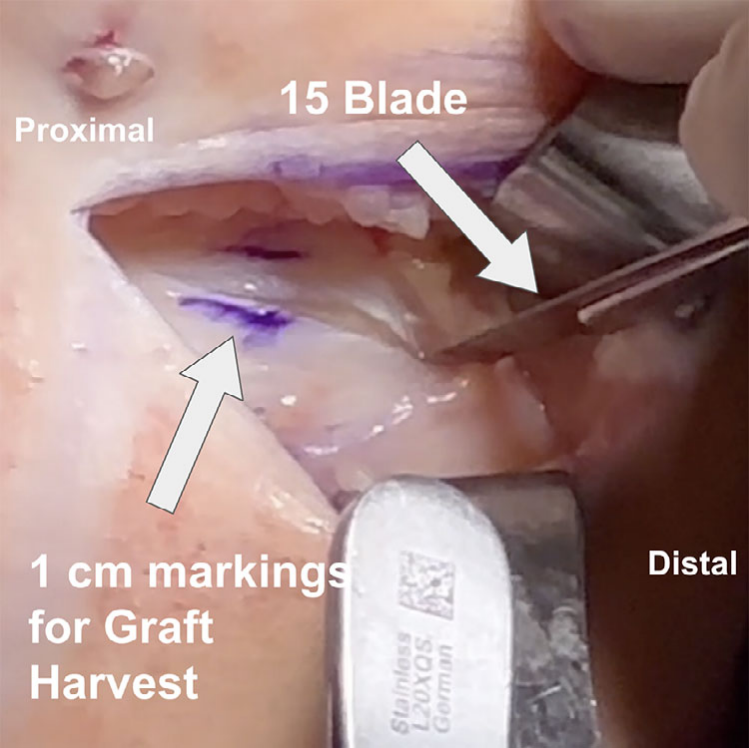
An open, lateral view of the right knee exposing the IT band. Before harvesting, 2 markings are made 1 cm apart along the center portion of the IT band. A 15 blade is then used to harvest the IT band while avoiding any damage to the deep structures beneath such as the LCL, the popliteus tendon, and the underlying capsule. (IT, iliotibial; LCL, lateral collateral ligament.)

**FIGURE 5:**
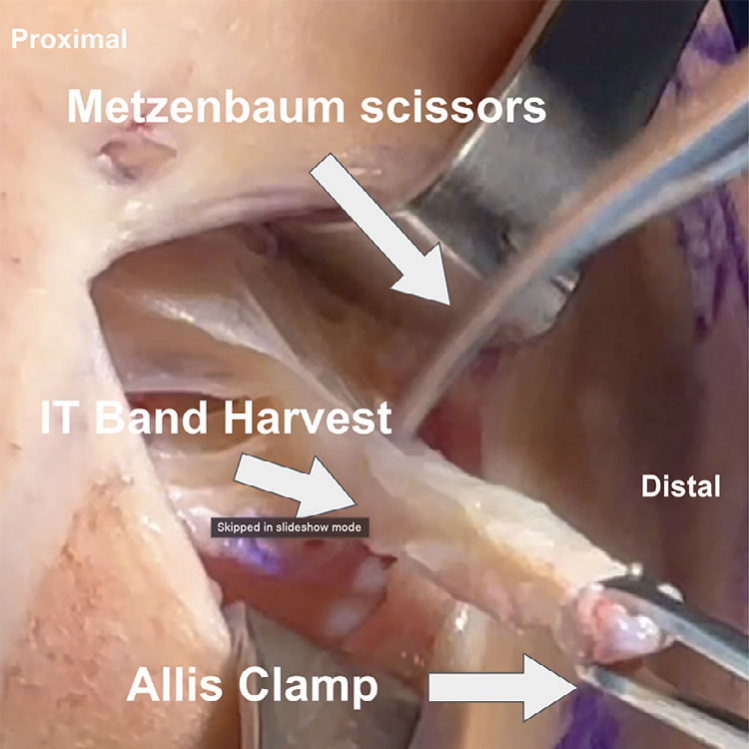
An open, lateral view of the right knee. Once the IT band graft length is confirmed with a ruler and the proximal end is transected, an Allis clamp is placed on the proximal end of the IT band. The distal portion of the IT band remains attached to Gerdy's tubercle. The underlying capsular attachments are then cleared with Metzenbaum scissors. (IT, iliotibial.)

#### LCL and Insertion Exposure

The knee is then placed at 90° of flexion and a slight figure‐of‐4 position. The LCL is located by palpation directly proximal to the fibular head. Metzenbaum scissors are used for sharp dissection at the anterior/distal aspect of the LCL to create an opening to the underlying capsule while protecting the LCL. Blunt dissection is then performed deep to the LCL, directed posteriorly or proximally with Metzenbaum scissors initially and then with a hemostat or right‐angle clamp. The combination of the 2 allows for access behind the LCL. A right‐angle clamp is used to confirm easy access, proximal to distal, deep to the LCL (Figure 6).

**FIGURE 6:**
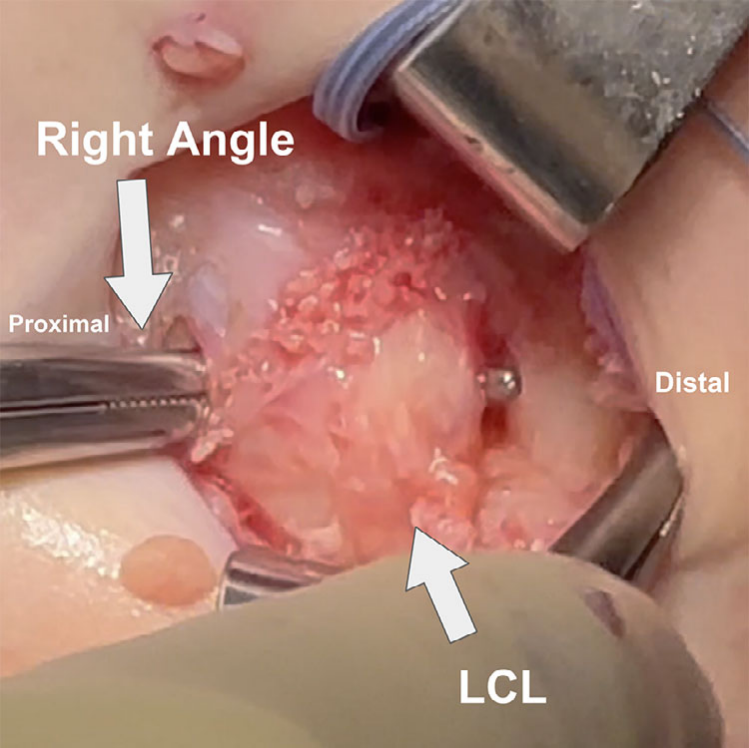
An open, lateral view of the right knee flexed to 90° and positioned in a slight figure‐of‐4 position. Sharp dissection is performed at the anterior/distal aspect of the LCL, while blunt dissection is performed posteriorly and proximally to create an opening deep to the LCL. A right angle is used to confirm easy access, proximal to distal, deep to the LCL. (LCL, lateral collateral ligament.)

### Suturing IT Band and Confirming LET Insertion

The IT band graft is whipstitched with 3 to 4 sutures (Smith & Nephew, Andover, MA) using a looped suture construct (Figure 7). The LET insertion is then confirmed as 8 mm proximal and 4 mm posterior to the LCL insertion. This is measured with a ruler to determine the appropriate anatomical landmarks prior to anchor placement.

**FIGURE 7:**
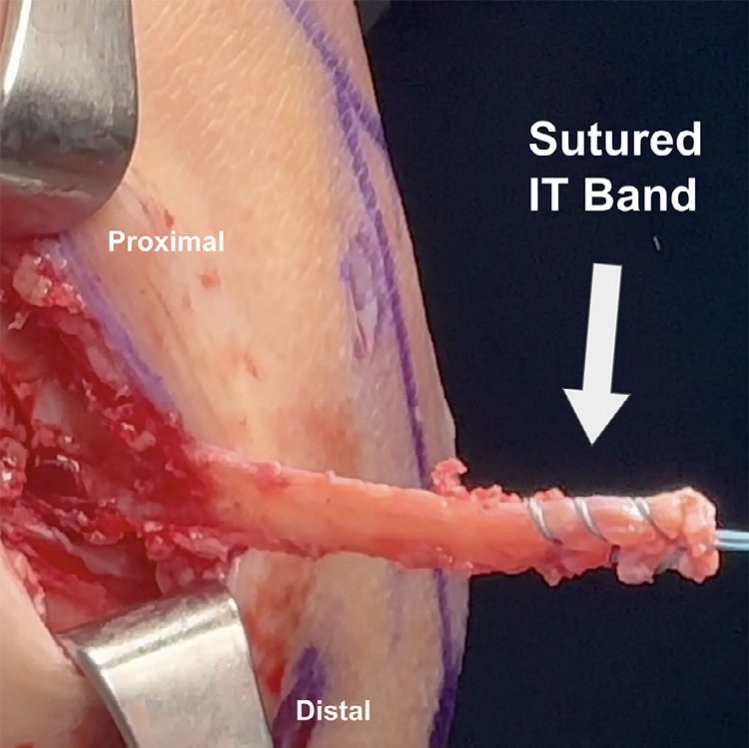
Right knee open, lateral view of the transected IT band. The IT band is whipstitched with 3‐4 sutures along the proximal end using a looped suture construct. (IT, iliotibial.)

Additional anatomic landmarks include the “leash of vessels,” which is adjacent to the lateral epicondyle. The graft fixation point is located 1.5 cm proximal and anterior to the “leash of vessels.” Another important landmark is the lateral supracondylar ridge, a bony prominence where the Kaplan fibers insert, located approximately 2 cm proximal and 1 cm posterior to the lateral epicondyle.

### Anchor Placement and Passing Graft

The drill guide (Smith & Nephew) is placed at the identified insertion site, and then, the femur is drilled proximally and anteriorly to avoid interference with any underlying tunnels or grafts. The 2‐mm drill is then advanced to a depth of 17.1 mm. The 1.8‐mm Q‐Fix knotless anchor (Smith & Nephew) is placed through the drill guide, preventing loss of trajectory (Figure 8). The Q‐Fix anchor is then deployed via a clockwise twisting mechanism of the anchor handle. The final width of the anchor, once fully deployed, can expand up to a maximum of 4 mm. Once the anchor is deployed, the IT band sutures are retrieved proximally, deep to the LCL. Placing the leg in valgus position allows for easier passage of the IT band graft (Figures 9 and 10). Once the graft is passed through, the graft is pulled proximally by the traction sutures, and the leg is flexed and extended, confirming isometry and point of fixation (Figure 11); the graft is then marked at this point.

**FIGURE 8:**
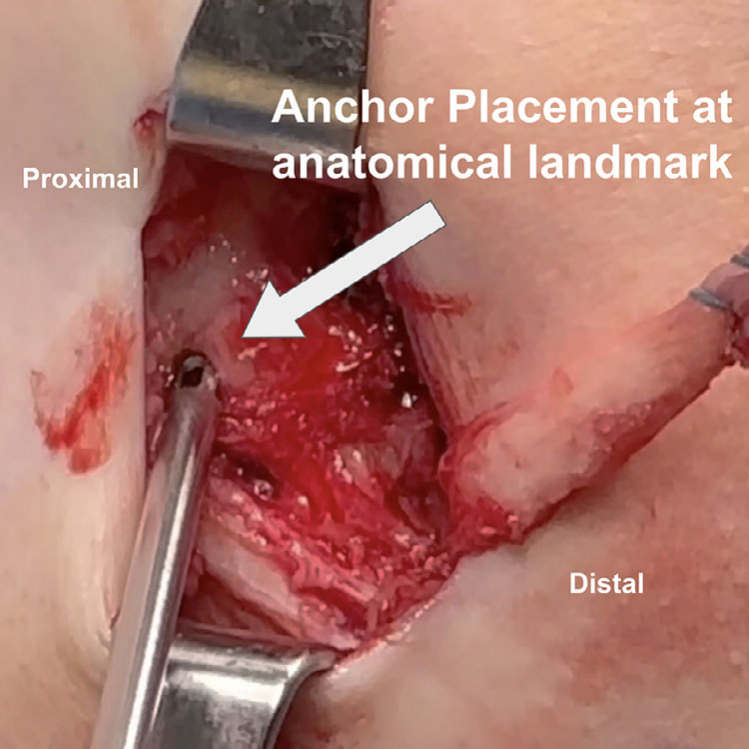
An open, lateral view of the right knee anchor placement. The femur is drilled proximally and anteriorly to avoid interference with the underlying tunnels or grafts from the ACL reconstruction at the identified insertion site. The Q‐Fix knotless anchor is then placed through the drill guide, preventing loss of trajectory. (ACL, anterior cruciate ligament.)

**FIGURE 9:**
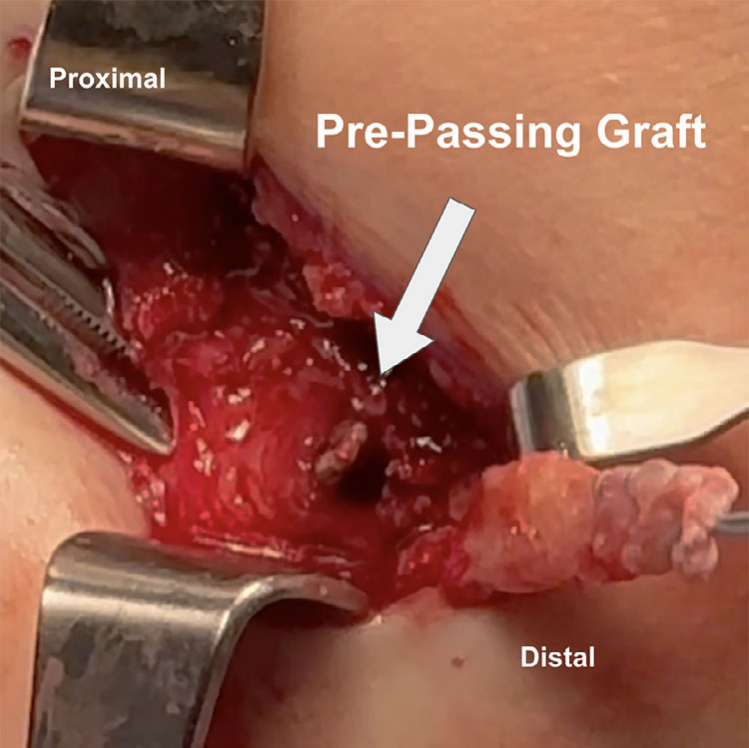
An open, lateral view of the right knee. A right angle is used to retrieve the IT band sutures proximally and deep to the LCL. (IT, iliotibial; LCL, lateral collateral ligament.)

**FIGURE 10:**
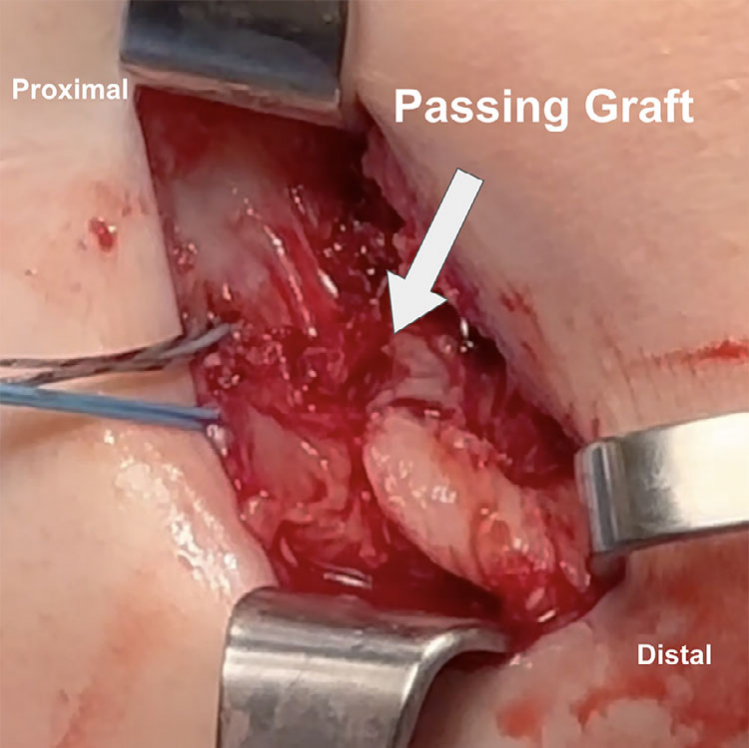
An open, lateral view of the right knee. The knee is placed in a valgus position to allow for easier passage of the graft.

**FIGURE 11:**
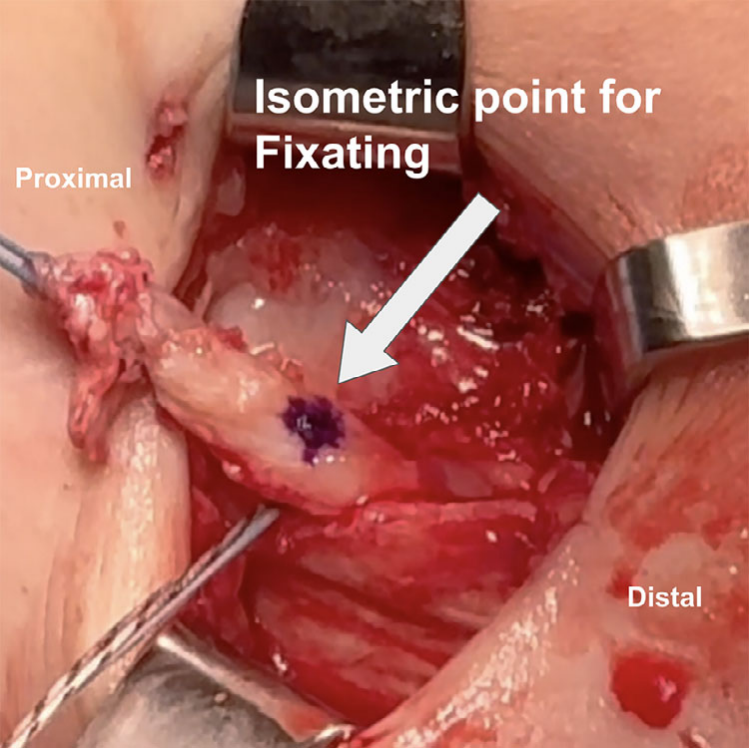
An open, lateral view of the right knee. Once the graft is passed through, it is then pulled proximally by the traction sutures. The leg is then flexed and extended to confirm it is isometric, and the point of fixation is marked.

### Fixating Graft and Loading and Deployment

The graft is whipstitched in a locking fashion 3 to 4 times with the blue leading suture/tape from the anchor (ULTRABRAID, Smith & Nephew). Once locked, the blue suture is placed through a loop at the end of the white passing suture from the Q‐Fix knotless anchor. Once loaded, the contralateral end of this passing suture is then pulled to deliver the blue suture into the knotless anchor, loading the anchor (Video 1). Recall that fixation occurs after ACL tensioning has been completed.

### Tensioning Anchor

Prior to final tensioning, the leg is placed at 20° of flexion with neutral rotation. Once in the appropriate position, final tensioning of the anchor is performed. The IT band autograft will reduce to the insertion site in an onlay fashion without any concern for tensioning, knot tying, socket interference, or hardware irritation (Figure 12). The IT band defect is closed with a No. 0 Vicryl suture and routine skin closure.

**FIGURE 12:**
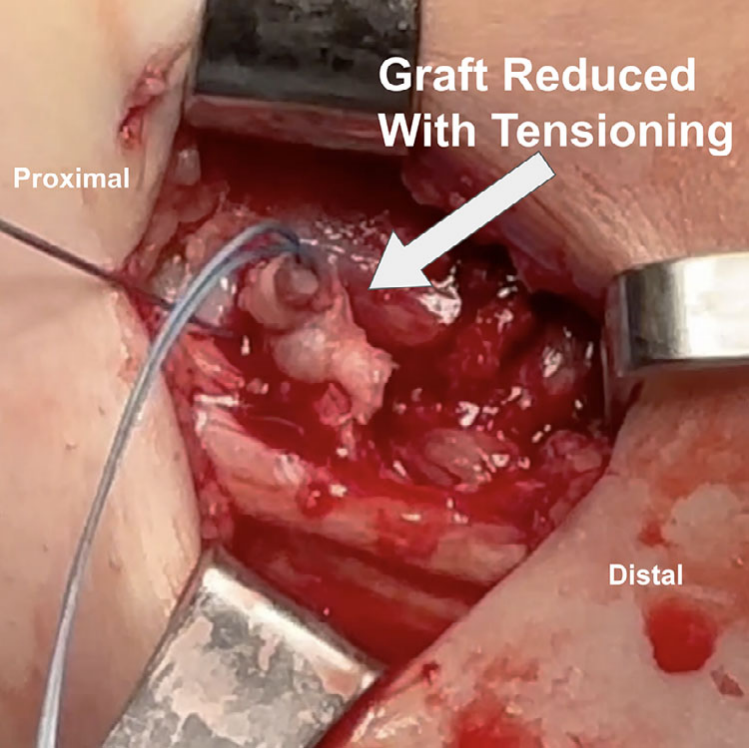
An open, lateral view of the right knee flexed at 20° with neutral rotation prior to final tensioning. Once in the appropriate position, the graft is reduced with a final tensioning of the anchor. On tensioning, the IT band autograft will be reduced in an onlay fashion. (IT, iliotibial.)

### Institutional Review Board Approval

IRB Tracking ID: 20183439.

### Postoperative Rehabilitation

The postoperative recovery is not adjusted for the modified LET procedure and follows standard ACLR postoperative restrictions and protocols. For ACLR without meniscal repair, patients will be weight‐bearing as tolerated with crutches. Patients will begin physical therapy, and full recovery is suspected around 1 year postop.

## DISCUSSION

LET augmentation in the setting of a primary or revision ACLR can be considered to increase rotational stability, decrease graft failure, and improve pivot‐shift grades compared with those who received ACLR only.^1^, ^3^ Persistent residual rotational instability puts the ACL graft under increased pressure, resulting in a higher risk of graft failure. LET augmentation reduces the stress placed on the ACL graft, supporting graft strength and maturation.^8^ Additional factors that may put patients at a high risk of persistent instability and possible ACLR failure include young age, return to cutting/pivoting sports postoperatively, and generalized ligamentous laxity.^9^ The efficacy of an LET augmentation in these populations has been proven.^1^, ^2^ This technical note describes a modified Lemaire LET using a 1.8‐mm all‐suture knotless anchor. One benefit of the described technique is the lack of interference with the femoral ACL fixation (Table 1). This all‐suture knotless anchor occupies a minimal footprint just deep to the lateral cortex, minimizing the risk of convergence with femoral ACL fixation or tunnel. The depth of the drill used for this anchor is 17.1 mm, which appears to be the most bone‐preserving and least likely to interfere with other hardware or tunnels compared with other published techniques.^10^ Given its subchondral position, the anchor generally does not cause soft‐tissue irritation.^9^ Finally, the knotless design of this anchor allows for a reproducible pull mechanism of tensioning the graft at the point of fixation in an “onlay” fashion. With traditional suture anchors, knot‐tying introduces an element of potential laxity. Similarly, with interference screw and socket fixation, there is a risk of under or overconstraint.^11^, ^12^

**TABLE 1:** Advantages and Disadvantages

**Advantages**	**Disadvantages**
Lack of interference of femoral ACL fixation because of the all‐suture knotless anchor occupying a minimal footprint deep to the lateral cortex.	Increased operative time can increase risks associated with surgical operation.
Harvest of the IT band autograft minimizes the need for additional graft site harvest.	Autologous IT band may provide graft site morbidity if the defect is not closed at the end of the procedure.

ACL, anterior cruciate ligament; IT, iliotibial.

In general, the modified Lemaire LET provides stability without the risk of osteoarthritis caused by overconstraint of the lateral compartment.^13^ Another benefit of the LET is the use of autograft tissue without detachment of the IT band from Gerdy's tubercle. This reduces the need for allograft tissue and additional tibial fixation as required in anterolateral ligament reconstructions.^9^ The LET procedure has also been used in the setting of a combined ACL and PCL reconstruction.^14^ Future studies are necessary to determine the outcomes of a combined multiligament reconstruction with LET.

The potential pitfalls and risks of this procedure should be noted (Table 2). Using the autologous IT band may provide graft site morbidity if the defect is not closed at the end of the procedure.^9^ Moreover, a posterior harvest puts an increased risk of bleeding and hematoma formation. Risks from increased operative time because of an additional augmentation procedure should also be noted. Other studies comparing functional outcomes between ACLR with and without an additional LET augmentation reported delayed recovery in the quadriceps to persist for 6 months. However, postoperative results between ACLR alone and ACLR with LET were similar.^15^

**TABLE 2:** Pearls and Pitfalls

**Pearls**	**Pitfalls**
Harvest the graft in the midportion of the IT band.	A posterior harvest of the IT band graft increases risks of bleeding and hematoma formation.
Palpate the LCL with the leg at 90° of flexion and use a fine right‐angle clamp to create the path deep to the LCL.	Do not incise the IT band too deep with the blade during harvest to prevent damage to the LCL.
Whipstitching the IT band graft and having a tapered end allow for easier passage.	Do not overconstrain the knee on tensioning. Apply neutral rotation with the knee at roughly 20° of flexion.
Passing the IT band graft with slight valgus to the knee allows for easier passage of the graft.	Avoid overtensioning of the graft to prevent overconstraint of the lateral compartment.
Always harvest a minimum of 8 cm of graft length.	

IT, iliotibial; LCL, lateral collateral ligament.

In summary, despite the wide variety of surgeon preferences for ACLR including graft choice, technique, and implants, comparatively fewer techniques and approaches have been described for LET augmentation. This technique provides an alternative method of performing an LET in the setting of an index or revision ACLR that is bone preserving, low‐profile, and reproducible.

## DISCLOSURES

The author (R.M.P.) declares the following financial interests/personal relationships which may be considered as potential competing interests: R.M.P. reports support for the present manuscript (e.g., funding, provision of study materials, medical writing, and article processing charges) from Smith & Nephew (consultant) and Vericel (consultant) and receives consulting fees. The other authors (L.L., S.T.) declare that they have no known competing financial interests or personal relationships that could have appeared to influence the work reported in this article.

## References

[1] Devitt BM , Bouguennec N , Barfod KW , Porter T , Webster KE , Feller JA . Combined anterior cruciate ligament reconstruction and lateral extra‐articular tenodesis does not result in an increased rate of osteoarthritis: A systematic review and best evidence synthesis. Knee Surg Sports Traumatol Arthrosc. 2017;25:1149‐1160. 10.1007/s00167-017-4510-1 28289822

[2] Lai S , Zhang Z , Li J , Fu WL . Comparison of anterior cruciate ligament reconstruction with versus without anterolateral augmentation: A systematic review and meta‐analysis of randomized controlled trials. Orthop J Sports Med. 2023;11:23259671221149403. 10.1177/23259671221149403 PMC10071203 37025126

[3] Boksh K , Sheikh N , Chong HH , Ghosh A , Aujla R . The role of anterolateral ligament reconstruction or lateral extra‐articular tenodesis for revision anterior cruciate ligament reconstruction: A systematic review and meta‐analysis of comparative clinical studies. Am J Sports Med. 2024;52:269‐285. 10.1177/03635465231157377 36960926

[4] Onggo JR , Rasaratnam HK , Nambiar M , et al. Anterior cruciate ligament reconstruction alone versus with lateral extra‐articular tenodesis with minimum 2‐year follow‐up: A meta‐analysis and systematic review of randomized controlled trials. Am J Sports Med. 2022;50:1137‐1145. 10.1177/03635465211004946 33886399

[5] Getgood A , Bryant D , Firth A , Stability Group . The stability study: A protocol for a multicenter randomized clinical trial comparing anterior cruciate ligament reconstruction with and without lateral extra‐articular tenodesis in individuals who are at high risk of graft failure. BMC Musculoskelet Disord. 2019;20:216. 10.1186/s12891-019-2589-x PMC6521537 31092226

[6] Smith PA , Thomas DM , Pomajzl RJ , Bley JA , Pfeiffer FM , Cook JL . A biomechanical study of the role of the anterolateral ligament and the deep iliotibial band for control of a simulated pivot shift with comparison of minimally invasive extra‐articular anterolateral tendon graft reconstruction versus modified Lemaire reconstruction after anterior cruciate ligament reconstruction. Arthroscopy. 2019;35:1473‐1483. 10.1016/j.arthro.2018.11.011 30926192

[7] Delaloye JR , Hartog C , Blatter S , et al. Anterolateral ligament reconstruction and modified Lemaire lateral extra‐articular tenodesis similarly improve knee stability after anterior cruciate ligament reconstruction: A biomechanical study. Arthroscopy. 2020;36:1942‐1950. 10.1016/j.arthro.2020.03.027 32251683

[8] Darville GL , Young BL , Lamplot JD , Xerogeanes JW . Arthroscopic‐assisted lateral extra‐articular tenodesis with knotless anchor fixation. Arthrosc Tech. 2023;12:e2257‐e2264. 10.1016/j.eats.2023.07.054 PMC10772996 38196854

[9] Temperato J , Ewing M , Nuelle CW . Lateral extra‐articular tenodesis with iliotibial band using knotless all‐suture anchor femoral fixation. Arthrosc Tech. 2023;12:e677‐e682. 10.1016/j.eats.2023.01.004 PMC10265525 37323783

[10] Haus A , Chand A , Dawson K , Lang S , Gilmer BB , Wahl CJ . Modified Lemaire lateral extra‐articular tenodesis using an inlay technique and all‐suture knotless anchor fixation. Arthrosc Tech. 2023;12:e1607‐e1613. 10.1016/j.eats.2023.05.004 PMC10533872 37780650

[11] Athiviraham A , Lee CS , Smith PA , Piepenbrink M , Mackin AP , Wijdicks CA . Self‐tensioning feature of knotless suture anchor provides reproducible knotless fixation independent of initial tension. Orthop J Sports Med. 2021;9:2325967121991593. 10.1177/2325967121991593 PMC8237213 34250166

[12] LeVasseur MR , Mancini MR , Williams N , et al. Ability to retension knotless suture anchors: A biomechanical analysis of simulated Bankart lesions. Orthop J Sports Med. 2022;10:23259671221098726. 10.1177/23259671221098726 PMC9208049 35734768

[13] Devitt BM , Bouguennec N , Barfod KW , Porter T , Webster KE , Feller JA . Combined anterior cruciate ligament reconstruction and lateral extra‐articular tenodesis does not result in an increased rate of osteoarthritis: A systematic review and best evidence synthesis. Knee Surg Sports Traumatol Arthrosc. 2017;25:1149‐1160. 10.1007/s00167-017-4510-1 28289822

[14] Gould HP , Zhuang ST , Marigi EM , Mayer J , Levy BA , Marx RG . Combined anterior cruciate ligament reconstruction, posterolateral corner reconstruction, and lateral extra‐articular tenodesis. Arthrosc Tech. 2025;14:103244. 10.1016/j.eats.2024.103244 PMC11977122 40207335

[15] Getgood A , Hewison C , Bryant D , et al. No difference in functional outcomes when lateral extra‐articular tenodesis is added to anterior cruciate ligament reconstruction in young active patients: The stability study. Arthroscopy. 2020;36:1690‐1701. 10.1016/j.arthro.2020.02.015 32147485

